# Real-time evaluation of longitudinal peak systolic strain (speckle tracking measurement) in left and right ventricles of athletes

**DOI:** 10.1186/1476-7120-7-17

**Published:** 2009-04-08

**Authors:** Laura Stefani, Gianni Pedrizzetti, Alessio De Luca, Roberto Mercuri, Gabriele Innocenti, Giorgio Galanti

**Affiliations:** 1 Sport Medicine Center, University of Florence, Florence, Italy; 2 Dept. Civil and Environmental Engineering, University of Trieste, Trieste, Italy

## Abstract

**Background:**

Strain, and particularly Longitudinal Peak Systolic Strain (LPSS), plays a role in investigating the segmental and overall contractility of the heart which is a particularly interesting feature in athletes in whom regular training determines several morphological and functional modifications in both the ventricles, that normally work at different loads. Speckle tracking techniques assess the LPSS of LV and RV from B-mode imaging in real time, with uniform accuracy in all segments, and can verify the possible dissimilar segmental contributions of the two chambers to overall myocardial contraction. The aim of the study is to quantify the LPSS in real time in both the ventricles in order to estimate any possible different deformation properties in them during a systolic period.

**Methods:**

32 subjects (20 athletes and 18 controls) were submitted to a standard echocardiographic examination at rest and after a Hand Grip (HG) stress. From a four-chamber-view image, the LPSS parameter was measured with Speckle Tracking analysis in the basal and medium-apical segments of the two ventricles, at rest and after HG.

**Results:**

In both athletes and controls, LPSS values were significantly higher in the RV of athletes (RV LPSS ^medium-apical ^-23.87 ± 4.94; ^basalfreewall ^-25.04 ± 4.12 at rest) and controls (RV LPSS^medium-apical ^-25.21 ± 4.97; ^basalfreewall ^-28.69 ± 4.62 at rest) than in the LV of both (athletes LV LPSS ^medium-apical ^-18.14 ± 4.16; ^basallateralwall ^-16.05 ± 12.32; controls ^medium-apical ^-18.81 ± 2.64; ^basallateralwall ^-19.74 ± 3.84) With the HG test a significant enhancement of the LPSS(with P < .05) in the medium-apical segments of LV and RV was evident, but only in athletes; there was no modification of the standard echo-parameters in either group.

**Conclusion:**

ST analysis is an easy method for investigating the contractility of the RV through deformation parameters, showing greater involvement of the RV than LV at rest. In athletes only, after isometric stress the two ventricles show particular myocardial deformation properties of the regions around the apex where the curvature of the wall is more marked. The clinical application of this new approach in athletes and normal subjects requires further investigation.

## Background

Regular sporting activity induces several morphological and functional modifications in all the heart chambers [1] and both the ventricles are involved in regularly-trained athletes. Currently, the Left Ventricle (LV) is usually more studied than the Right Ventricle (RV), which normally presents several difficulties for non-invasive examination.

Normally the RV is considered a low-charge and therefore "passive" chamber, and its function has usually been assessed in a bi-dimensional echo exam by measurement of Right Ventricle Area Change (RVAC). This parameter has been found to correlate best with MRI-derived Right Ventricle Ejection Fraction (RVEF) [2]and to be a specific and sensitive indicator mainly when RV dysfunction is severe [3], while it gives poor and incomplete information on segmental contractility when the RV function is normal, as in healthy subjects or athletes.

An innovative investigation of overall and segmental heart performance is now possible, using new parameters such as quantification of strain (S) on the walls [4-6]. S is a physically -based measure of myocardial deformation and is considered to be a useful tool for studying the systolic function in physiological hypertrophy [7]. Two methods, one based on Tissue Doppler analysis (TDI)and the other on grey scale Speckle Tracking (ST) are currently available for determining the S. They are consistent methods when applied at the basal levels of the myocardial chambers [8], but only the latter method, which is angle-independent, can evaluate the S with high reproducibility in the rest of the ventricular regions, including the medium- apical segments.

Of the deformation parameters currently used, Longitudinal Peak Systolic Strain (LPSS) is one of the most common and validated for determining global and segmental myocardial contractility in normal subjects and in athletes [9-11]

In athletes, the LPSS measurement has recently been proposed to complete the evaluation of LV myocardial function at rest and after a brief isometric stress [10,11]. On the other hand, few studies have investigated the role of the S parameter as a new ultrasound index for quantifying the performance of the RV chamber. The currently-available data mainly regard the child population affected with congenital cardiac disease [12,13], and only one study has evaluated the effects on RV performance [14] of a prolonged endurance sport activity like marathon, demonstrating a transient decrease in strain values.

The aim of the present study is to quantify LPSS in the basal and medium- apical segments of the RV chamber in real time in a group of regularly-trained athletes, and to compare these values with the LV LPSS ones in order to verify any possible difference between them. For this reason, the ST method was preferred, since its angle independence can be applied to all segments of the two ventricles, including the regions with the greatest curvature like the wall segments near the apex. In order to highlight any possible dissimilar properties of the single segments in the deformation of the two ventricles analyzed, a brief isometric exercise, the Hand Grip (HG) test, was introduced into the study protocol.

## Methods

### Subjects

This study included 50 subjects: 32 competitive athletes aged 25 ± 8.1 years who regularly trained at high dynamic and low-moderate static component [15] three times a week for at least 2 hours for almost six months per year, matched with 18 healthy sedentary subjects as controls. All of them gave their oral informed consent before participating in the study, which had been approved by Institutional Ethic Committee.

All were submitted to a general check-up to exclude cardiovascular, metabolic or familiar diseases.

### Study protocol

The study protocol was divided into two phases: an echocardiographic examination at rest, and re-measurement of echo parameters at the peak of the HG test. The HG test consisted of an isometric stress test performed with a graduated handle dynamometer held in the dominant arm for three minutes The handle dynamometer (baseline hydraulic hand dynamometer No. 10533 USA) was regulated at 30% of the previously- established maximum effort. A short, acute effort such as HG was chosen in order to create a pressure load enhancement in both ventricle chambers and thus in the deformation of the myocardial wall. During both the rest and stress phases, a clip of an echo-image in the four-chamber view, including three beats, was captured for each subject in order to perform strain analysis.

Systolic and Diastolic Blood Pressure (SBP, DBP) and heart rate (HR) were measured both at rest and at the end of the acute effort.

### Echocardiographic data acquirement

All the echo-studies were performed with the subjects lying in a supine position. The images were acquired using the My Lab 30 echocardiograph equipped with a 2.5 MHz probe, and later processed with the X-Strain software for evaluating 2D strain. The traditional echo parameters, including Left Ventricle End Diastolic diameter (LVEDd), Left Ventricle End Systolic Diameter (LVESd), thickness of Inter Ventricular Septum (IVS) and Posterior Wall (PW), were obtained from a long-axis view and according to AHA guidelines [16]. Ejection Fraction (EF) was determined starting from a four-chamber view employing Simpson's rule method, from end-systolic and end-diastolic endocardial border traces. Cardiac Mass Index (CMI g/m2) was calculated from the Devereux procedure [17]. The evaluation of diastolic function was performed using the transmitral flow, calculating E-wave and A-wave velocities, Isovolumic Relaxation Time (IVRT), and Deceleration Time (DT). Two images from a four-chamber view, one at rest and the second at the peak of effort, were acquired for further investigation of deformation parameters. In order to exclude the effect on RV deformation parameters of the varying increase in Pulmonary Pressure (PP) after the HG test, the mean PP was indirectly calculated in a short-axis view, from the pulmonary outflow Doppler acceleration time [16] at rest and after HG. With the image samples captured in the four-chamber view at rest and with HG, the RVAC of each subject was calculated from the equation:



[8]. All the echocardiographic data were averaged over three beats.

### Longitudinal Strain measurement by speckle tracking model

Images were acquired with the subjects at rest, lying in the lateral decubitus position with data acquisition at the expiration stage so as not to create "artefacts". The 2D images of four-chamber views were post-processed with X-Strain software to provide an angle-independent tool for the evaluation of velocities and strain. This software allows automatic evaluation of the dynamic properties of the endocardial border and of the sub-endocardial tissue from 2D B-mode echocardiographic clips [11]. Strain analysis with ST is independent of translational motion, tethering effects of the nearby regions, and it thus allows uniformity of measurements through the normal LV myocardium. The endocardial border is drawn by the operator in a four-chamber view on a single frame from one annulus to another; the first and last points delineate the mitral plane. The LPSS was measured in basal and mid-apical segments of the LW and IVS from the images captured at rest.

Thanks to the angle-independence, the LPSS measurements were performed in basal and medium-apical segments of the RV free lateral wall and LV lateral wall of the images acquired at rest and after HG (Additional file 1, Image 1, Additional file 2, image 2, Additional file 3, clips 1, Additional file 4, clip 2).

This procedure was performed in double-blind by two expert operators with a week's interval in between and following the same protocol. All the echocardiographic analyses were performed without any knowledge of the results of the reference methods. To assess inter-observer variability, 6 echocardiographic tests were randomly selected and then independently analyzed by two different observers with the same method.

### Statistical analysis

All the data were expressed as mean ± Standard Deviation (SD). Statistical and power analysis were performed using Statview and STATA (Stata – Corp 2003) software. The comparisons among athletes and controls at rest and at peak stress were performed with Student's two-tailed unpaired test, while the comparison between basal (resting) state and Hand Grip for each group was performed with Student's paired "t" test. We considered results significant at a value of p < 0.05. The inter-observer variability was calculated as the SD of differences in ST strain measurements by two independent observers for 6 randomly-selected echocardiographic examinations.

## Results

### General and Echocardiographic data

**Figure 1:**
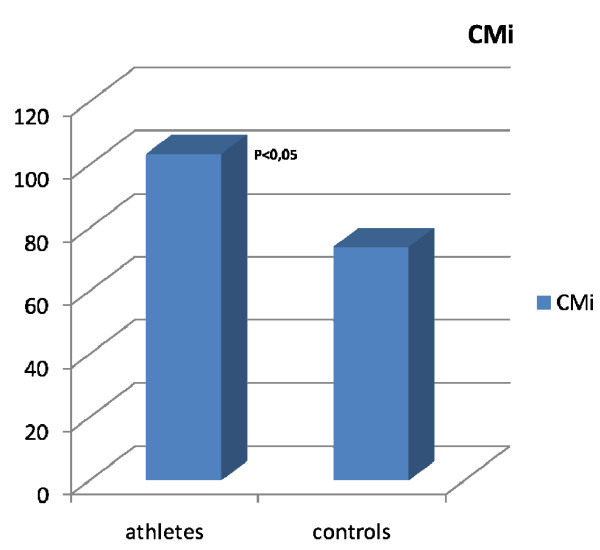
**The figure shows the significant difference of CMi in athletes and controls**.

**Figure 2:**
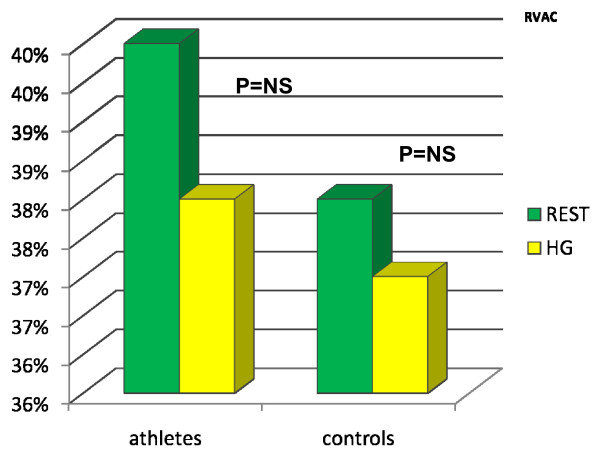
**The figure shows the increase in athletes and controls of the RVAC (Right Ventricle Area Change) values from the rest to the HG state**.

**Figure 3:**
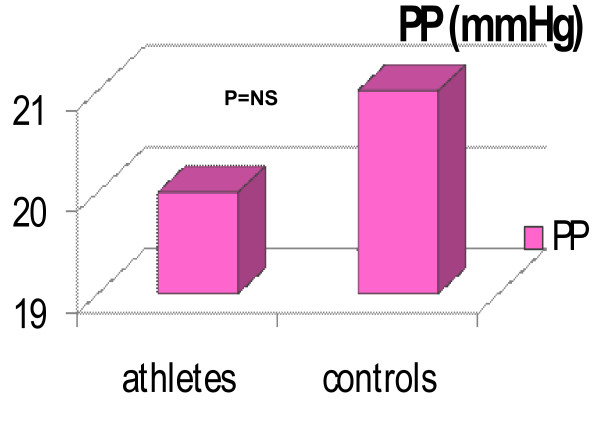
**The figure shows the progressive increase of PP in athletes and controls with HG test**. The PP values are maintained within the normal range in both and the difference between the two groups are non significant.

The general and standard echocardiographic data at rest of the two groups studied is collected in Table 1. All the parameters considered were similar in the two groups with the exclusion of CMI, which was significantly higher in athletes than in controls (Tab 1, Fig 1). After HG, the Ejection Fraction (EF) showed a physiological increase in both groups (EF ^athletes ^up to 63%, EF ^controls ^up to 64%). The RVAC showed a mild but non-significant decrease at the end of the HG test in both groups (RVAC^athletes ^up to 0.40%; RVAC^controls ^up to 0.38%; Fig 2). PP mean values remained within the normal range at rest (Tab 1) and after HG (PP ^athletes ^up to 22 ± 3 mmHg after HG; PP ^controls ^up to 20 ± 3 mmHg after HG. Fig 3). The mean systolic and diastolic Blood Pressure (BP) values showed a significant enhancement in athletes and controls with the HG test (in athletes it increased up to 140/90 mmHg, in controls up to 145/85 mmHg). Heart Rate (HR) was slightly higher than basal values after HG in both groups (athletes reached 88 ± 4 bpm, controls reached 89 ± 2 bpm) (see table 1).

**Table 1:** Echocardiographic and general findings of controls and athletes at rest

***Controls***		***Athletes***	
BSA	22.64 ± 3.5	23.18 ± 4	NS
HR (bpm)	70 ± 10	60 ± 5	NS
SBP mmHg	130 ± 5	120 ± 3.4	NS
DBP mmHg	70 ± 4.5	60 ± 4	NS
LA mm	31.07 ± 3.57	36.90 ± 4.43	NS
Aorta mm	30.975 ± 3.64	28.54 ± 3.26	NS
LVDD mm	50.37 ± 4.11	51.63 ± 4.43	NS
LVSD mm	32.25 ± 3.53	28.45 ± 4.18	NS
CMI gr/m^2^	92 ± 12.5	119.23 ± 10	p < .05
IVS mm	8.43 ± 0.93	9.6.55 ± 1.2	NS
PW mm	8.35 ± 0.89	9.64 ± 1.3	NS
RVAC %	0.39 ± 3.4	0.40 ± 4.5	NS
EF%	60%	61%	NS

BSA: Body Surface Area; HR: Heart Rate; SBP: Systolic Blood Pressure; DBP: Diastolic Blood Pressure; LA: Left Atrium; LVDD: Left Ventricular Diastolic Diameter; LVSD: Left Ventricular Systolic Diameter; CMI: Cardiac Mass Index: IVS: Inter Ventricular Septum; PW: Posterior Wall; RVAC: Right Ventricle Area Change.

### Longitudinal Peck Systolic Strain findings

The LPSS values of RV were significantly higher both at rest (athlete RV LPSS basal free wall -25.04 ± 4.12 and medium-apical -23.87 ± 4.94; control RV LPSS basal free-wall -28.69 ± 4.62 and medium -apical -25.21 ± 4.97) and after HG (athlete RV LPSS basal free wall -24.16 ± 7.38 and medium-apical-26.63 ± 3.72; control RV LPSS basal free-wall -25.28 ± 3.56 and medium -apical -28.15 ± 5.52) than in LV both at rest (athletes LV LPSS basal lateral wall -16.05 ± 12.32 and medium-apical -18.14 ± 4.16; controls LV LPSS basal lateral wall-19.74 ± 3.84 and medium apical -18.81 ± 2.64) and after HG (athlete LV LPSS basal lateral wall -18.46 ± 4.52 and medium apical -24.07 ± 7.51; control LV LPSS basal lateral wall -20.68 ± 3.64 and medium apical -19.91 ± 5.15).

**Figure 4:**
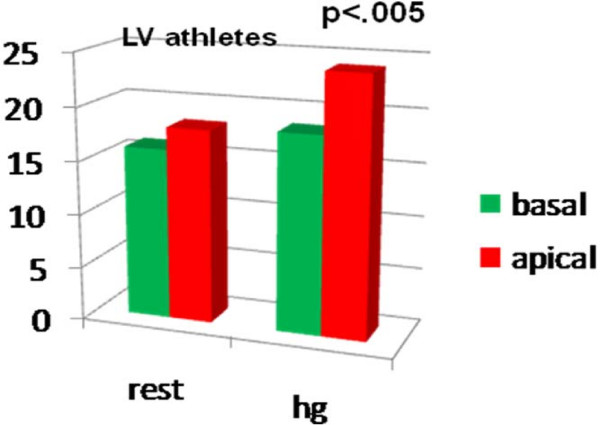
**Longitudinal Peak Systolic Strain in basal and medium-apical segments of LV at rest (Left side) and after HG (right side) in athletes.**.

**Figure 5:**
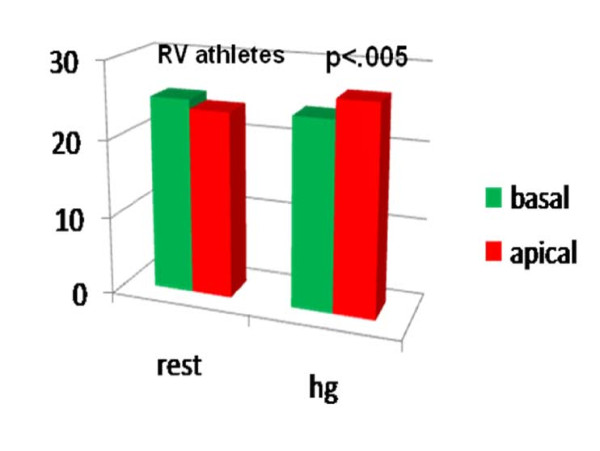
**Longitudinal Peak Systolic Strain in basal and medium-apical segments of RV at rest (left side) and after HG (right side) in athletes**.

**Figure 6:**
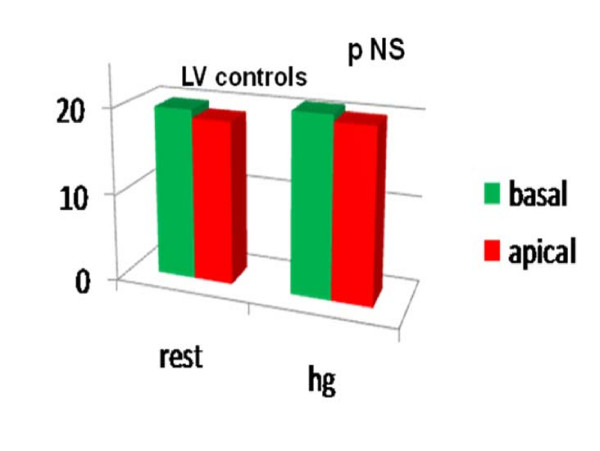
**Longitudinal Peak Systolic Strain in basal and medium-apical segments of LV at rest (left side) and after HG (right side) in controls.**.

**Figure 7:**
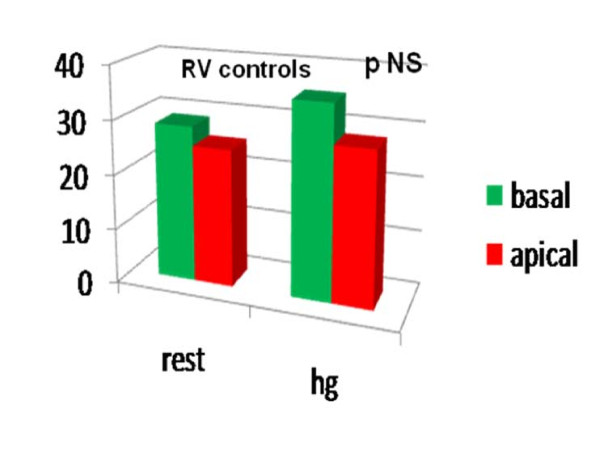
**Longitudinal Peak Systolic Strain in basal and medium-apical segments of RV at rest (left side) and after HG (right side) in controls.**.

Comparing the LPSS values of the basal and medium- apical segments of the two ventricles at the end of the HG test, we noted a significant LPSS increase in the medium-apical segments of the left and right chambers (LV LPSS ^medium-apical ^from -18.14 ± 4.16 at rest to -24.07 ± 7.51; RV LPSS ^medium-apical ^from -23.87 ± 4.94 at rest to -26.63 ± 3.72 with HG; P < .05 for all) in the athlete group alone. (Table 2, Fig 4, Fig 5). Conversely, in controls, a mild, non-significant increase in RV LPSS was evident at rest in basal as compared with medium apical segments (basal free-wall -28.69 ± 4.62 vs -25.21 ± 4.97 in medium apical) and the HG test showed a clear enhancement which was not however sufficient to indicate a significant increase (basal free-wall -25.28 ± 3.56 up to -28.15 ± 5.52) (Fig 6, Fig 7). Regarding the other echo parameters evaluated, in the RV (RVAC and PP) no evident changes were present in either group, although there was a slight tendency to decrease. The inter-observer variability for ST strain was 5.8% (p = NS) (See table 2).

**Table 2:** LPSS values in LV and RV of athletes and controls

		***Athletes***			***Controls***		
		***rest***	***HG***	***P***	***rest***	**HG**	***P***
***LV***	Basal lateral wall	-16.05 ± 12.32	-18.46 ± 4.52	*NS*	-19.74 ± 3.84	-20.68 ± 3.64	*NS*
	Med- apical	-18.14 ± 4.16	-24.07 ± 7.51	< .05	-18.81 ± 2.64	-19.91 ± 5.15	*NS*
***RV***	Basal free wall	-25.04 ± 4.12	-24.16 ± 7.38	*NS*	-28.69 ± 4.62	-25.28 ± 3.56	*NS*
	Med- apical	-23.87 ± 4.94	-26.63 ± 3.72	< .05	-25.21 ± 4.97	-28.15 ± 5.52	*NS*

LPSS (Longitudinal Peck Systolic Strain) LV (Left Ventricle); RV (Right Ventricle); HG (Hand Grip); Basal (basal segments) Med-apical(medium apical segments).

## Discussion

Previous studies have focused on the importance to evaluate the athlete's heart performance with the measurement of LPSS [10,11] in addition to the standard echo-parameters. Particularly in athletes, respect of controls, the interest to study in real time the two ventricles is due to the fact that LV and RV are both involved in the morphological and functional modifications. Currently data on the LV chamber deformation properties of healthy athletes after a brief isometric stress [10] are presented, while no information is yet available on RV performance, except for a study where a persistent but reversible decrease in LPSS, calculated by the TDI method, has been demonstrated after endurance sport activity [14].

In the present study, an evaluation of the deformation properties of the athlete's RV by the ST LPPS calculation has been, for the first time, considered. The results obtained show that all the LPSS values of the RV are significantly higher in both basal and medium-apical segments of athletes (-25.04 ± 4.12; -23.87 ± 4.94) and controls (-28.69 ± 4.62; -25.21 ± 4.97) than in the LV of both groups (athletes -16.05 ± 12.32; -18.14 ± 4.16; controls -19.74 ± 3.84; -18.81 ± 2.64). Despite the data are in agreement with the results previously found in healthy young normal subjects [9], nonetheless they present some differences in the values of the LPSS in the basal segment of the RV lateral wall, which in our study are higher than the validated range in normal subjects. It is reasonable to think that the different software used to measure the strain value in the previous study, based on TDI and therefore angle-dependent, could go some way to explaining the dissimilar values with a tendency to strain underestimation. Even though the two methods are in fact consistent when applied to the basal segment of the ventricles [8], the quantification of strain by TDI can often be influenced by overall heart motion and contraction in adjacent segments.

Considering that heart contractility in the young athletes investigated matched that of the healthy sedentary controls subjects, the former seem to maintain a trend similar to the latter when they are in the same conditions. However in the RV of athletes, as in the LV, isometric stress produces a peculiar deformation involving the medium-apical segments, with higher negative values of LPSS, and in the presence of unchanged RVAC. The same behavior is not recognizable in controls.

In addition, the non-significant modification of the PP values found in both groups after isometric stress confirm that RV strain performance in athletes does not directly depend on PP variation. Probably a particular morphology and function of the RV chamber of athletes as compared to normal subjects, despite the divergent pressure- work-load of the two ventricles, might be involved in the different level of their deformation properties. Thus the Speckle Tracking method can be helpful to distinguish the two different levels of contractility of both chambers in athletes vs normal subjects even if the evaluation of the intimate properties of the myocardial fibers is outside the scope of the present investigation. Nonetheless it is reasonable to suppose that several clinical applications will be found for this pioneering non- invasive approach in athletes. It may be possible to discover the early stages of dissimilar or deficient contractility in the presence of an asymptomatic myocardial or valve disease where an involvement of one of the two ventricles is more evident.

## Limits of the study

The study includes a restricted number of subjects due to the need to have a homogeneous group. We must thus consider the importance of enrolling and evaluating larger numbers of controls and athletes who are matched for BSA and age. It is reasonable to think that this might be one of the reasons for some dissimilar strain values found in this study. Despite this limitation, the purpose to use real-time investigation of LV and RV myocardial strain could be acceptable if we consider the possibility of several other clinical implications of this approach in patients and athletes where the early stages of impairment of heart performance are not yet associated with evident symptoms [11].

## Conclusion

These results, in agreement with the previous data in literature [7,9,10], lend additional validity to the ST method for real-time evaluation of myocardial deformation properties in athletes. Despite the low number of subjects analyzed in the present investigation, the data obtained do suggest the important role of the RV chamber in overall myocardial contractility in athletes. The measurement of the LPSS can better separately characterize the performance of the two ventricles in an athlete's heart and immediately distinguish their different functions which seem to be particular and different from those of the normal subject.

## Competing interests

The authors declare that they have no competing interests.

## Authors' contributions

The initial idea for the study was of LS. LS, GG designed the study, and LS, GG, GP, NM, ADL performed all the measurements and statistical analyses. LS wrote the manuscript and all the authors contributed to, read, and approved the final version.

## Supplementary Material

Additional File 1 **Image 1**. Image 1: Image of "feature tracking" calculation of LPSS (using X-Srain) in left ventricle, including one systolic cycle at rest. All peak strain values are represented as negative. A different colour curve corresponds to the LPSS value of the single myocardial segment. For example in this section the purple curve corresponds to the strain value in the basal segment of LV lateral wall, while the white curve for example corresponds to the LPSS of the interventricular septum. Click here for file

Additional File 2 **Image 2**. Image of "feature tracking" calculation of LPSS (using X-Strain) in right ventricle, including one systolic cycle at rest. All peak strain values are represented as negative. A different colour curve corresponds to the LS value of the single myocardial segment. The red curve represents the LPSS in the medium apical segments of the free wall of RV while the orange curve is an example of the application corresponding to the LPSS measurement in the interventricular septum. The same procedure can be applied during stress. Click here for file

Additional File 3 **Clips 1**. This clip shows the use of X-Strain software to obtain measurement of the LPSS in all the segments of the LV chamber during a systole. Click here for file

Additional File 4 **Clip 2**. This clip shows the use of X-Strain software to calculate the LPSS in all the segments of the RV during a systole. Click here for file
