# Supplementary figures and images for: Real-time evaluation of longitudinal peak systolic strain (speckle tracking measurement) in left and right ventricles of athletes
Source: Cardiovasc Ultrasound. 2009 Apr 8;7:17. doi: 10.1186/1476-7120-7-17 (PMC2676253; doi:10.1186/1476-7120-7-17)

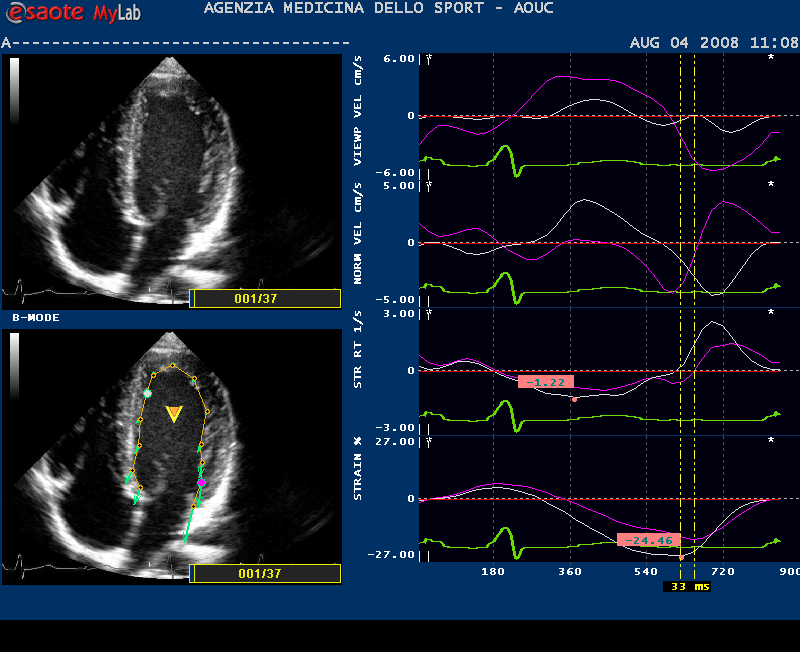

Supplement: Additional File 1 — Image 1. Image 1: Image of "feature tracking" calculation of LPSS (using X-Srain) in left ventricle, including one systolic cycle at rest. All peak strain values are represented as negative. A different colour curve corresponds to the LPSS value of the single myocardial segment. For example in this section the purple curve corresponds to the strain value in the basal segment of LV lateral wall, while the white curve for example corresponds to the LPSS of the interventricular septum. [file 1476-7120-7-17-S1.bmp]

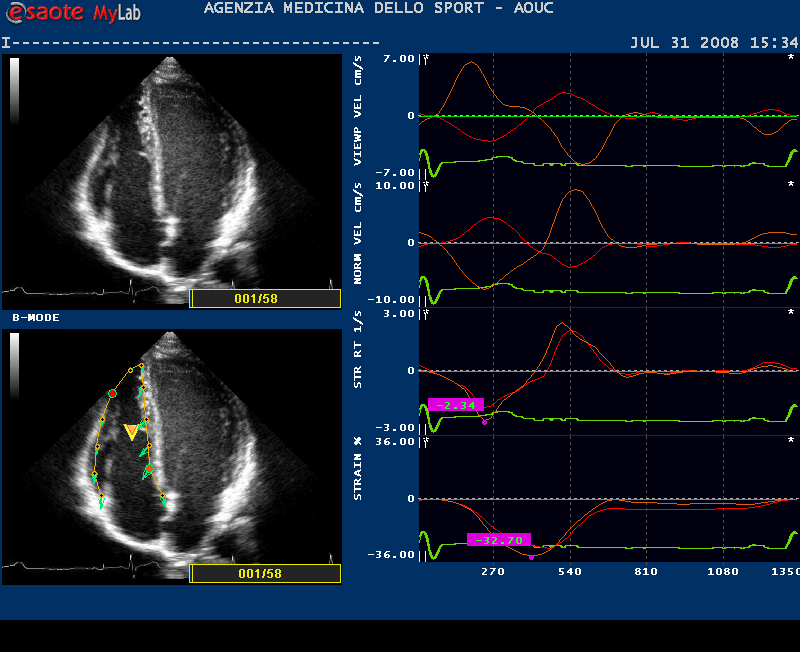

Supplement: Additional File 2 — Image 2. Image of "feature tracking" calculation of LPSS (using X-Strain) in right ventricle, including one systolic cycle at rest. All peak strain values are represented as negative. A different colour curve corresponds to the LS value of the single myocardial segment. The red curve represents the LPSS in the medium apical segments of the free wall of RV while the orange curve is an example of the application corresponding to the LPSS measurement in the interventricular septum. The same procedure can be applied during stress. [file 1476-7120-7-17-S2.bmp]
